# A novel large intragenic *DPYD* deletion causing dihydropyrimidine dehydrogenase deficiency: a case report

**DOI:** 10.1186/s12920-024-01846-2

**Published:** 2024-03-25

**Authors:** Anna Malekkou, Marios Tomazou, Gavriella Mavrikiou, Maria Dionysiou, Theodoros Georgiou, Ioannis Papaevripidou, Angelos Alexandrou, Carolina Sismani, Anthi Drousiotou, Olga Grafakou, Petros P. Petrou

**Affiliations:** 1 Biochemical Genetics Department, The Cyprus Institute of Neurology and Genetics, P. O. Box 23462, 1683 Nicosia, Cyprus; 2 Bioinformatics Department, The Cyprus Institute of Neurology and Genetics, P. O. Box 23462, 1683 Nicosia, Cyprus; 3 Cytogenetics and Genomics Department, The Cyprus Institute of Neurology and Genetics, P. O. Box 23462, 1683 Nicosia, Cyprus; 4 Department of Pediatrics, Inborn Errors of Metabolism Clinic, Archbishop Makarios III Hospital, Korytsas 6, 2012 Nicosia, Cyprus

**Keywords:** *DPYD*, Dihydropyrimidine dehydrogenase, Fluoropyrimidines, Copy number variants, Deletion, Toxicity

## Abstract

**Background:**

Dihydropyrimidine dehydrogenase (DPD), is the initial and rate-limiting enzyme in the catabolic pathway of pyrimidines. Deleterious variants in the *DPYD* gene cause DPD deficiency, a rare autosomal recessive disorder. The clinical spectrum of affected individuals is wide ranging from asymptomatic to severely affected patients presenting with intellectual disability, motor retardation, developmental delay and seizures. DPD is also important as the main enzyme in the catabolism of 5-fluorouracil (5-FU) which is extensively used as a chemotherapeutic agent. Even in the absence of clinical symptoms, individuals with either complete or partial DPD deficiency face a high risk of severe and even fatal fluoropyrimidine-associated toxicity. The identification of causative genetic variants in *DPYD* is therefore gaining increasing attention due to their potential use as predictive markers of fluoropyrimidine toxicity.

**Methods:**

A male infant patient displaying biochemical features of DPD deficiency was investigated by clinical exome sequencing. Bioinformatics tools were used for data analysis and results were confirmed by MLPA and Sanger sequencing.

**Results:**

A novel intragenic deletion of 71.2 kb in the *DPYD* gene was identified in homozygosity. The deletion, *DPYD*(NM_000110.4):c.850 + 23455_1128 + 8811del, eliminates exons 9 and 10 and may have resulted from a non-homologous end-joining event, as suggested by in silico analysis.

**Conclusions:**

The study expands the spectrum of *DPYD* variants associated with DPD deficiency. Furthermore, it raises the concern that patients at risk for fluoropyrimidine toxicity due to *DPYD* deletions could be missed during pre-treatment genetic testing for the currently recommended single nucleotide polymorphisms.

**Supplementary Information:**

The online version contains supplementary material available at 10.1186/s12920-024-01846-2.

## Background

Dihydropyrimidine dehydrogenase (DPD; EC 1.3.1.2) catalyzes the reduction of the pyrimidine bases, uracil and thymine to 5,6-dihydrouracil and 5,6-dihydrothymine, respectively [1]. DPD deficiency (MIM #274270) results in the accumulation and increased urinary excretion of uracil and thymine that can be detected during basic metabolic work-up [2]. It is estimated that approximately 3–8% of individuals in the Caucasian population display partial DPD deficiency [3], whereas complete deficiency is much rarer with an estimated incidence of 0.1–0.3% [4]. The clinical presentation of subjects with DPD deficiency displaying thymine-uraciluria varies largely and ranges from asymptomatic individuals to patients with early-onset neurological manifestations which include recurrent seizures, microcephaly, muscular hypotonia, intellectual disability, motor and mental retardation and autistic behaviour [5].

DPD is also the main metabolizer of 5-fluorouracil (5-FU), which belongs to a group of anticancer drugs termed fluoropyrimidines (FPs) and are widely used in the treatment of a broad range of solid tumors, most commonly gastrointestinal, breast and head as well as neck cancers [6]. FPs (5-fluorouracil, its oral prodrug capecitabine and other analogs) are the most prescribed cytostatic drugs for solid cancers, and approximately 2 million cancer patients worldwide are treated with this class of chemotherapeutics each year [7]. Approximately 80% of the administered 5-FU is rapidly eliminated by DPD through its conversion to the inactive metabolite 5, 6-dihydro-5-fluorouracil in the liver [8]. Individuals with DPD deficiency, even those exhibiting no clinical symptoms, are therefore susceptible to severe and potentially life-threatening 5-FU-associated toxicity. Around 20–30% of patients treated with FPs experience severe toxicity which can be fatal in up to 1% of the cases, with DPD deficiency being the main cause [9]. Because of this, the European Medicines Agency (EMA) has recommended testing for DPD deficiency before patients start chemotherapy with FPs either by measuring pre-treatment plasma levels of uracil or by genetic testing for specific *DPYD* variant alleles [10].

The *DPYD* gene (NG_008807.2) is located on chromosome 1p21.3 and spans 850,3 kb (GRCh37/hg19) out of which only 3078 bp are coding [11, 12]. *DPYD* is organized into 23 exons ranging between 69–961 bp, which are surrounded by large intronic regions with an average size of 43 kb [12]. Currently, more than 400 single nucleotide variants are listed in ClinVar, however, only a small number of these have been classified as pathogenic/likely pathogenic or have been functionally linked with 5-FU toxicity. Clinical studies have established four *DPYD* variant alleles: c.1679 T > G p.(Ile560Ser) (rs55886062), c.2846A > T p.(Asp949Val) (rs67376798), c.1129–5923C > G (rs75017182) and c.1905 + 1G > A (rs3918290) to be associated with increased risk for 5-FU toxicity [13, 14]. Whereas the incorporation of this single nucleotide variant (SNVs) genotyping into clinical practice can avoid 25–50% of severe cytotoxic effects [15], additional rare *DPYD* variants have been associated with 5-FU-related toxicity, as listed in the Pharmacogene Variation Consortium (PharmVar) database (www.PharmVar.org) [16]. Several of these variants, were found to result in partial or total loss of DPD activity after functional experiments in an isogenic mammalian system [17]. Moreover, advances in high-throughput technologies in molecular diagnosis such as next generation sequencing, led to the identification and characterization of novel rare variants such as c.2087G > A p.(Arg696His) and c.2324 T > G p.(Leu775Trp) [16]. Copy number variations (CNVs) have also been associated with DPD enzyme deficiency. Recently, a novel intragenic deletion of *DPYD* exon 4 resulting in a truncated DPD enzyme (p.Cys79Thrfs*8) was detected at high prevalence in Finnish cancer patients [18]. At least 20 additional independent CNVs in *DPYD* have been reported. These include both partial and whole gene deletions as well as whole duplications [19], suggesting that in addition to SNVs, CNVs could comprise an appreciable risk factor for DPD deficiency-related 5-FU toxicity.

In the present study, we report a novel homozygous intragenic large deletion of 71.2 kb encompassing exons 9 and 10 of the *DPYD* gene identified in a Cypriot male infant patient displaying thymine-uraciluria.

## Methods

### Patient

A male infant, the first offspring of non-consanguineous healthy Greek-Cypriot parents, born at 39 + 5 weeks of gestation with a birth weight of 2640 gr, was referred for clinical evaluation due to failure to thrive, frequent vomiting, and diarrhea, not responding to different formulas. He was a smiling alert infant with signs of dermatitis, willing to take his hypoallergic formula on which he was gaining appropriate weight. The psychomotor development of the infant was normal. The clinical picture was compatible with milk protein allergy and gastroesophageal reflux which was later confirmed by 24 h pH-metry. During metabolic work-up, the patient was found to repeatedly exhibit increased levels of methionine [272 μmol/L (reference range: 5–55 μmol/L)] and homocysteine [12 μmol/L (reference range: 3.3–8.3 μmol/L)] in plasma, which normalized when the formula was changed to one with reduced methionine content. Moreover, urine organic acid analysis revealed prominent thymine-uraciluria, suggesting a genetic defect in pyrimidine catabolism.

### Urine Organic Acid Analysis

Qualitative urine organic acid analysis was performed by gas chromatography-mass spectrometry (GC–MS) on an Agilent 6890N/5973 system, essentially as described in [20]. Briefly, following oximation with hydroxylamine hydrochloride, organic acids were extracted from urine samples using liquid–liquid (ethylacetate and ether) extraction, converted to their corresponding trimethylsilyl ethers with BSTFA + 1% TMCS and subjected to chromatographic separation. The identity of organic acids was determined by comparing the generated mass spectra to a custom library.

### Clinical Exome Sequencing (CES)

Genomic DNA was extracted from peripheral blood samples obtained after informed consent using the MagCore Genomic DNA Whole Blood kit (RBC Biosciences) and an automated extractor. DNA libraries for CES, were prepared using the TruSight One sequencing panel (Illumina, San Diego, CA, USA). The panel includes the following genes implicated in pyrimidine metabolism: *DHODH*, *UMPS*, *NT5C3*, *TYMP*, *DPYD*, *DPYS*, *UPB1*, *TK2* and *CDA*. A complete list of the genes included in the panel can be found in https://www.illumina.com/products/by-type/clinical-research-products/trusight-one.html. Paired-end sequencing of the pooled libraries was performed on a NextSeq 500 system (Illumina) using the High Output Kit v2.5 (300 Cycles) according to the manufacturer’s guidelines (Illumina, San Diego, CA, USA). Demultiplexing and adapter trimming was performed automatically using BaseSpace Sequencing Hub Apps (Illumina, San Diego, CA, USA). Bioinformatics processing, analysis, annotation and interpretation was performed with the VarSome Clinical platform (Version: 11.4) using the human reference genome build hg19. Variants were classified according to the guidelines of the American College of Medical Genetics and Genomics (ACMG) (https://www.acgs.uk.com/quality/best-practice-guidelines/) and the recommendations of the Association for Clinical Genomic Science (ACGS) (https://clinicalgenome.org/working-groups/sequence-variant-interpretation), assisted by VarSome’s automated ACMG classifier.

### Copy number variation (CNV) analysis

All fastq files from all samples of the specific CES run were processed using a local installation of the nf-core/Sarek v3.0.2 [21] pipeline built in the nextflow framework [22]. Reads underwent quality control in FastQC v0.11.9 [23] and fastp v0.23.2 [24] for low quality base call filtering and adapter trimming. The filtered reads were aligned against the GRCh37 reference genome and the resulting alignments (in.bam format) were marked for duplicates and recalibrated using the GATK’s Baserecalibrator module [25]. Mosdepth v.0.3.3 [26] was used to obtain base-resolution coverage depth of the targeted regions, as listed in the Illumina’s Trusight One CES V1 manifest (https://support.illumina.com/downloads/trusight_one_sequencing_panel_product_file.html). Next, the CNVkit v0.9.9 [27] was used in batch mode to normalize coverages across the entire run and calculate the corresponding log2 coverage ratio for each sample. Finally, the CNVkit call function was used for copy number calling at each probe locus for each sample. IGV.js [28] was used to generate visualisations of the loci of interest, while processing and visualisation of the raw coverages and CNVkit calls was performed using custom written scripts in R programming language [29] and packages tidyverse [30] and ggplot2 [31].

### Multiplex ligation-dependent probe amplification (MLPA) analysis

MLPA was performed using the SALSA MLPA probemix P103-C1 DPYD (MRC Holland), according to the manufacturer’s guidelines. This probemix contained 45 MLPA probes, including 33 probes for exons, two probes specific for the c.1129-5923C > G and the c.1905 + 1G > A (IVS14 + 1G > A) variants and two probes specific for the wild type sequence of the c.1679 T > G and the c.2846A > T variants. Additionally, nine quality control probes were included. The MLPA PCR products were run on an Applied Biosystems 3500XL Genetic analyzer using the GeneScan 500 LIZ Size Standard (Thermo Fisher Scientific) and the data were analysed with the Coffalyser software (MRC Holland).

### PCR and Sanger sequencing

A total of 14 primer sets were designed against the reference sequence of the *DPYD* gene (NG_008807.2, NM_000110.4) to cover introns 8 (83928 bp, 9 sets) and 10 (19247 bp, 5 sets) producing amplicons of approximately 500 bp. The patient’s and a control DNA were screened by PCR for the presence or absence of regions targeted by these primer sets. PCR amplification was carried out using Amplitaq Gold DNA polymerase (Applied Biosystems, Thermo Fisher Scientific). Long range PCR was performed using FastGene Taq 2 × Ready Mix (Nippon Genetics) according to the manufacturer’s protocol. The final amplification product of 3 kb was run on a 0.8% agarose gel and purified by gel extraction (NucleoSpin Gel and PCR Clean-up Kit, Μacherey-Νagel). Sanger sequencing was performed on the purified PCR product using the BigDye Terminator v1.1 Cycle Sequencing Kit (Applied Biosystems, Thermo Fisher Scientific). The sequencing reactions were cleaned-up using the Performa® DTR Gel Filtration Cartridges (EdgeBio, Gaithersburg, MD, USA) and capillary electrophoresis was performed on an Applied Biosystems 3500XL Genetic Analyzer. The sequencing data were compared to the normal *DPYD* sequence as listed in the GenBank database.

Primers used for PCR amplification and Sanger sequencing are listed in Supplementary Table 1.

### In silico analysis of sequences at the deletion breakpoints

Screening of DNA sequences for interspersed repeats and low complexity sequences was performed with the RepeatMasker program (http://repeatmasker.genome.washington.edu) using “cross_match” as a search engine and sequences of 1000 bp upstream and downstream of each breakpoint. The search for potential non-B DNA secondary structures, such as triplexes, quadruplexes, hairpin/cruciforms, Z-DNA and single-stranded looped-out structures was performed inside the region of 100 bp both upstream and downstream of the deletion breakpoints by the Non-B DNA Motif Search Tool (https://nonb-abcc.ncifcrf.gov/apps/nBMST/default/). The presence of palindromic sequences at a distance of < 50 bp from the breakpoints was performed using the EMBOSS tool (https://www.bioinformatics.nl/cgi-bin/emboss/palindrome). The BDGP NNsplice software (https://fruitfly.org/seq_tools/splice.html) was used as a splice site predictor.

## Results

**Fig. 1:**
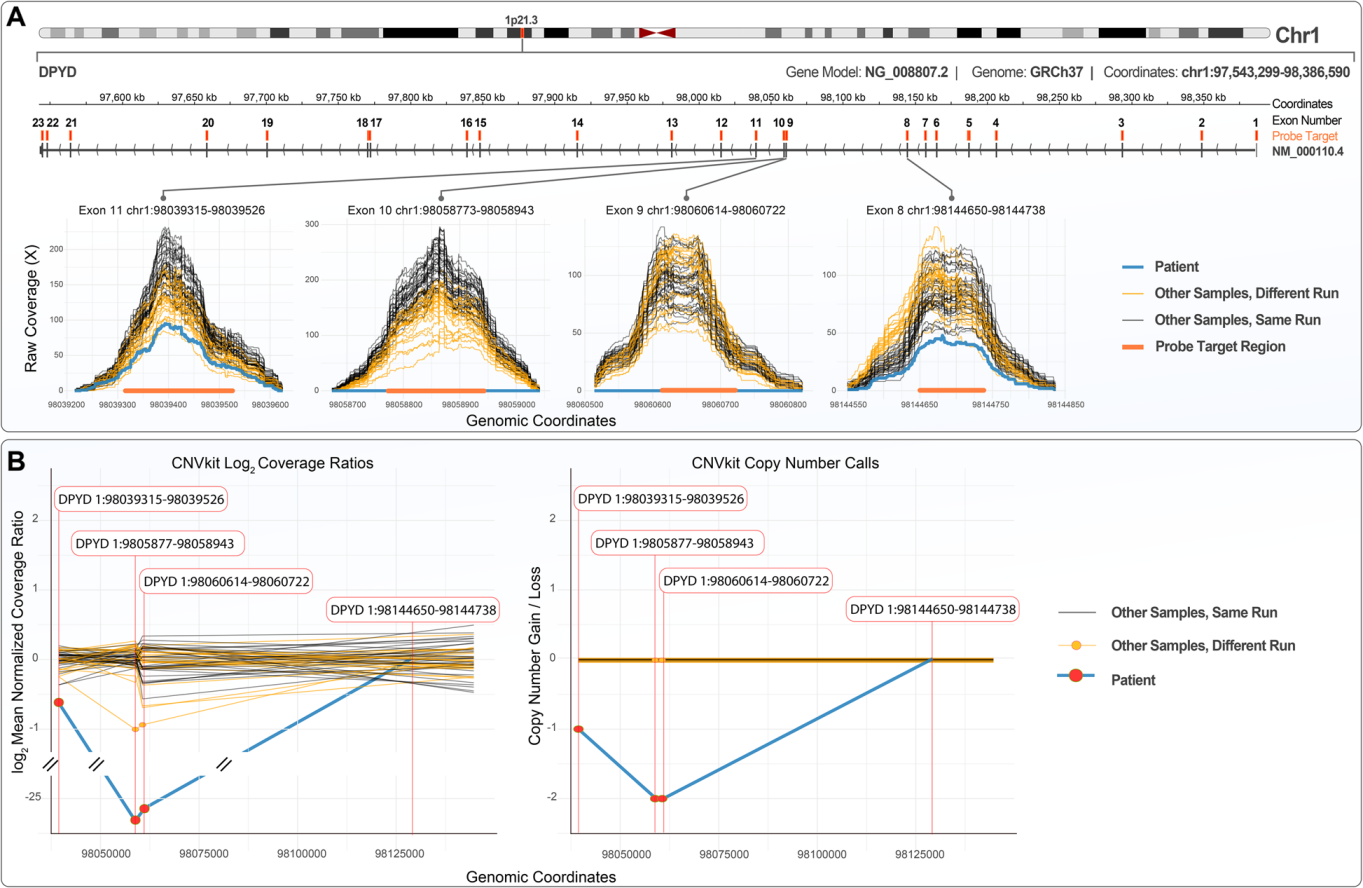
Coverage- and copy number variation analysis of *DPYD* exons. **A** Top: Ideogram of chromosome 1 and schematic representation of the *DPYD* gene located at the 1p21.3 locus and the NM_000110.4 transcript. Exons are illustrated as vertical lines, regions targeted by probes as orange rectangles and introns as horizontal lines with arrows. Arrows denote the direction of transcription. Bottom: Raw coverage analysis plots for exons 8–11 of *DPYD*, showing no coverage of exons 9 and 10 in the patient (blue colour) in contrast to the other samples of the same CES run (yellow colour) and those of a different CES run (black colour). **B** Copy number variant (CNV) analysis plots. Left plot illustrates the log2 coverage ratios after normalization across all samples of each run [same CES run (yellow lines), different CES run (black lines)]. The right plot shows the copy number calls for each probe for each sample. In both plots the blue line represents the patient’s sample

Increased excretion of thymine and uracil is the biochemical hallmark of DPD deficiency but is also compatible with additional enzyme defects such as dihydropyrimidinase- (dihydropyrimidinuria; MIM #222748) and thymidine phosphorylase deficiency [mitochondrial DNA depletion syndrome 1 (MNGIE type); MIM #603041]. To identify the underlying genetic aberration, we performed Clinical Exome Sequencing (CES), which did not identify any causative single nucleotide variants (SNVs) or small insertions-deletions (INDELs) in genes implicated in pyrimidine metabolism. Particularly, for *DPYD*, *DPYS* and *TYMP* encoding dihydropyrimidine dehydrogenase, dihydropyrimidinase and thymidine phosphorylase, respectively only benign variants were detected. We next questioned the presence of large structural variants (e.g. large insertions or deletions) in *DPYD, DPYS* and *TYMP*. To this end, we performed a coverage-based CNV analysis using the NGS coverage depth at the loci of interest. The analysis revealed that *DPYD* exons 9 and 10 (NM_000110.4) had no coverage (0X depth), which is indicative of a homozygous deletion (Fig. 1A). To assess the overall quality and completeness of the targeted *DPYD* region, short read coverage-based CNV analysis was performed using all 36 samples from the same CES run and 24 additional samples from a different CES run. These were random samples from symptomatic patients investigated for a genetic disorder. The analysis revealed that in contrast to the patient’s all other samples were fully covered (~ 100% coverage at depth > 20X) demonstrating that the hybridization probes provided adequate representation of the specific loci in the NGS library (Fig. 1A). Consistent with the above, the CNVkit analysis following coverage normalization for all samples, returned a deletion call for the specific exons of the patient (Fig. 1B).

**Fig. 2:**
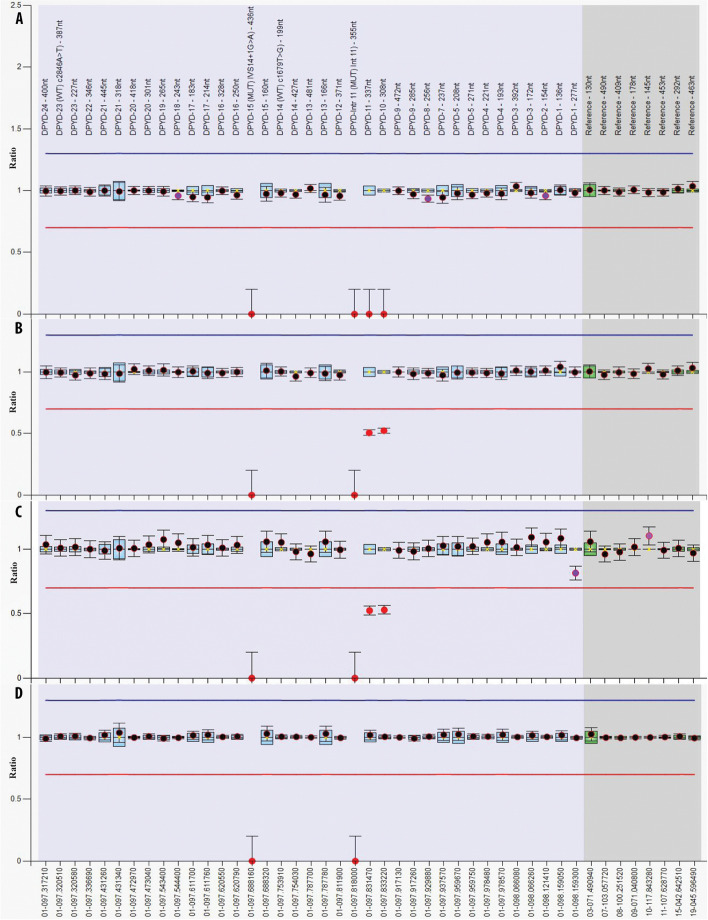
Analysis of copy number variation in the *DPYD* gene using MLPA. MLPA ratio charts of the patient (**A**), the patient’s mother (**B**), the patient’s father (**C**) and a healthy individual used as control (**D**). For the analysis, 33 probes covering exons, four probes within *DPYD* introns (two specific for the wild type sequence and two for the c.1129-5923C > G and the IVS14 + 1G > A variants, respectively) as well as 9 control, reference probes (coloured in grey) were used. The blue and red lines represent the cut-off values, used to determine increased or reduced copy numbers of targeted sequences. Note that the annotation of exons in the SALSA probemix used for MLPA analysis takes into account an additional exon (designated as Exon 6) which is not present in transcript NM_000110.4 and for which no probe was included. Hence, the missing exons in the present analysis are indicated as Exons 10 and 11

Subsequent assessment of *DPYD* exon copy number by MLPA confirmed the presence of a deletion in homozygosity in the patient (Fig. 2A) and at a heterozygous state in both parents (Fig. 2B-C). As indicated by the probes 05326-L04713 and 05327-L04714 the deletion eliminates exons 9 and 10, respectively (Fig. 2A-D).

**Fig. 3:**
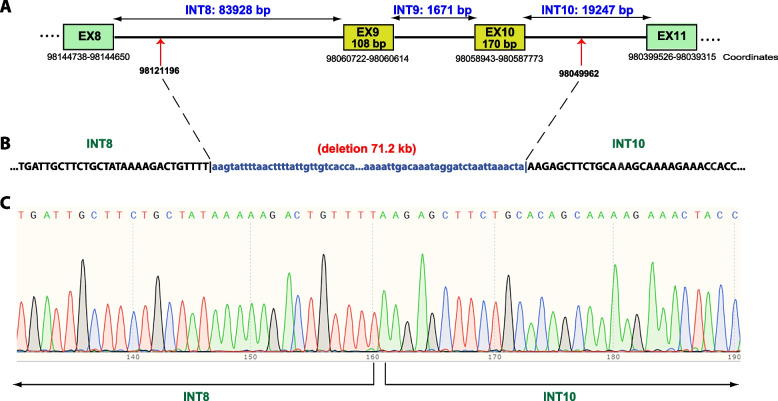
Characterization of the breakpoints of the *DPYD* deletion. **A** Schematic illustration of part of the *DPYD* transcript variant NM_000110.4. Exons are represented by rectangles and intervening introns by lines. Red arrows denote the position of the breakpoints. **B** Nucleotide sequence, upstream and downstream of each breakpoint. The deleted sequence is indicated in blue letters. **C** Sequencing electropherogram of the patient at the junction region

Exons 9 and 10 (NM_000110.4) are 108 bp and 170 bp, respectively and the intervening intron is 1671 bp in length. As suggested by MLPA analysis, the breakpoints of the identified deletion could lie anywhere within the flanking introns 8 and 10, which are 83928 bp and 19247 bp, respectively (Fig. 3A). Based on the above, the *DPYD* deletion has a potential size of 104.4 kb. To further delineate the deleted region, a PCR-based approach was employed to confirm the presence or absence of DNA segments at both ends. Ultimately, using a forward primer in intron 8 and a reverse primer in intron 10 in a long-range PCR, a single product of about 3 kb was obtained in the patient. Following Sanger sequencing of this amplicon the deletion was found to span a region of 71.2 kb with the 5’ breakpoint lying 23455nt downstream of exon 8 and the 3’ border 8811nt downstream of exon 10 (Fig. 3).

The presence of repetitive elements such as long- and short interspersed nuclear elements (LINEs and SINEs) including Alu sequences, as well as DNA structures (e.g. non-B conformation DNA and palindromes) was assessed around the breakpoints using bioinformatics tools. No repetitive SINEs, including Alu sequences were detected near the breakpoints (Supplementary Table 2). However, several LINE/L1 repeats were identified at the 3’ breakpoint within intron 10. In particular, two LINE/L1 (L1P1) repeats were found to lie exactly at this breakpoint, one located upstream (824-1000nt) and the other downstream (1-611nt) (Supplementary Table 2) whereas no LINE/L1 retrotransposons were present at the 5’ breakpoint within intron 8. Additionally, no relevant non-B DNA motifs or palindromic sequences were detected in close proximity to the breakpoints and no microhomology was observed at the breakpoint junction. Overall, the in silico analysis may suggest non-homologous end joining (NHEJ) as a possible mechanism for the formation of the identified *DPYD* c.850 + 23455_1128 + 8811del variant.

## Discussion

In the present study we report a novel intragenic *DPYD* deletion variant, c.850 + 23455_1128 + 8811del of 71.2 kb [Chr1:98,049,962-98121196del (GRCh37)], identified in a patient with biochemical features characteristic of DPD deficiency. The above variant has not been previously reported in DECIPHER (https://www.deciphergenomics.org), ClinVar (https://www.ncbi.nlm.nih.gov/clinvar/) or the Database of Genomic Variants (DGV) (http://projects.tcag.ca/variation). The deletion was identified after coverage-based CNV analysis which highlights the limitations of the currently available NGS platforms to detect large deletions. This is mainly due to the generation of millions of short reads (50-300 bp) that can be frequently misaligned or a reference pool data set that lacks quality (uniformity and depth of coverage), making the detection of large deletions challenging.

As predicted by the NNsplice tool, the identified variant does not seem to generate a new splice donor or acceptor site at the position of the breakpoints. The deletion is predicted to cause a shift in the reading frame resulting in the incorporation of incorrect amino acids at positions 284–287, followed by a premature termination codon. Whether this predicted truncated protein variant is stable and actually synthesized is currently unknown. The deleted amino acid region eliminates the most important and functional domains of the enzyme: the NADP^+^ binding domain (amino acids 335 – 487), which is essential for its catalytic activity, the second FAD binding domain (442 – 524), the FMN binding domain (532 – 834) that also includes the pyrimidine binding site (609 – 737) and the two C-terminal 4Fe-4S clusters (848 – 1025) [32]. Hence, even if this truncated protein variant is stably expressed it would lack structural and functional domains essential for DPD enzymatic activity. Although enzyme activity measurements are required for confirmation, the prominent thymine-uraciluria present in the patient is in support of severely impaired DPD activity associated with the identified deletion.

A large number of *DPYD* SNVs are listed in the ClinVar database. However, for the vast majority, the pathogenicity and clinical significance is uncertain. Despite the considerable variations with regards to the clinical presentation, most patients with DPD deficiency display neurological symptoms, whereas no clear genotype–phenotype correlation has so far been established [5]. In addition to SNVs, several CNVs have been identified in *DPYD*. These, mostly include either exon- or whole gene deletions ranging between 10–120 kb and 1.1–14 Mb, respectively [19]. Patients with CNVs appear to share common clinical symptoms which include intellectual disability, autism-like features and speech delay [19]. However, the majority of the reported CNVs affect other genes in addition to *DPYD*, therefore the clinical phenotype cannot be solely attributed to DPD deficiency. The herein described patient, currently at the age of 1 year, does not present any neurological symptoms. A potential association of the herein described deletion with clinical manifestations can be established by the frequent neurological monitoring of the patient.

Interestingly, the herein described *DPYD* deletion lies within the FRA1E fragile site which is one of the well characterized common fragile sites that are listed in the Genome database. The above fragile site extends over *DPYD* exons 9–18 with exons 13–16 corresponding to the region displaying the highest fragility [33]. Common fragile sites can give rise to genomic alterations such as translocations, duplications and deletions [34] and may play a role in the generation of *DPYD* CNVs. CNVs arise by different mechanisms which include repair-associated processes of double-strand DNA breaks (DSBs), such as non-allelic homologous recombination (NAHR), non-homologous end joining (NHEJ), fork stalling and template switching (FoSTeS), and microhomology-mediated replication-dependent recombination events (MMRDR) [35]. Repetitive elements such as long- and short interspersed elements (LINEs and SINEs) including Alu sequences, as well as DNA structures (e.g. non-B conformation DNA and palindromes) are typically enriched around breakpoints and can trigger the formation of DSBs [36, 37]. For the deletion reported in this study, no microhomology regions or sequence motifs prone to double strand break formation were identified using bioinformatics tools. This may suggest non-homologous end joining as a likely mechanism for the formation of this particular *DPYD* deletion.

It is well established that DPD deficiency is a major determinant of toxicity associated with FP chemotherapy [4, 9]. Aiming in improving the efficacy versus toxicity ratio for patients, the prevalence of *DPYD* variants and their association with 5-FU-induced toxicity was assessed in different populations [38, 39]. Following the release of the EMA recommendations in 2020 [10], testing for DPD deficiency either biochemically by measuring plasma uracil levels or by genotyping specific *DPYD* SNP risk alleles has significantly increased in many European countries [40]. Nevertheless, despite genotyping, more than 20% of patients carrying none of the four *DPYD* risk variants still display adverse effects related to FP treatment [41, 42], suggesting the existence of additional risk alleles.

CNVs, in addition to SNVs, have been long overlooked but are currently increasingly being recognized as important genetic determinants of drug response. It has been reported that *DPYD* deletions account for approximately 7% of DPD deficiency cases [43]. Moreover, an intergenic *DPYD* deletion which includes exon 4 has recently been identified in 4 out of a cohort of 167 Finnish patients who were scheduled to initiate FP therapy [18]. All heterozygous carriers of the above deletion displayed decreased DPD activity, within the range of heterozygous carriers of known pathogenic SNVs [18]. The above study demonstrates that a significant number of DPD-deficient individuals eligible for FP chemotherapy is not picked up by genotyping only for the recommended four most common, clinically relevant variants, and faces an increased risk of potentially lethal toxicity. Testing for well-characterized CNVs in addition to the recommended *DPYD* SNP variants may therefore improve screening sensitivity. An important question arising is whether *DPYD* deletions, including the herein reported variant, are population specific or also present in different ethnic groups. Interestingly, the exon 4 deletion identified in Finnish patients has recently been reported in 1 out of 250 patients of a Canadian cohort [19]. An additional deletion of 13.8 kb eliminating *DPYD* exon 12 was identified in patients of different ethnic ancestry [43]. Screening for population-specific variants might therefore be a more effective way of identifying patients at risk. A search in an in-house database containing array-based comparative genomic hybridization (array-CGH) data identified the herein reported deletion in heterozygosity in one out of 900 individuals. In a total of 960 samples investigated the estimated allele frequency in the Cypriot population is 0.0005. We acknowledge the lack of power and specificity in the estimation of the allele frequency of the identified deletion in the Cypriot population as a limitation of this study. Due to the lack of a national/ethnic variation database, our estimation was mainly based on the analysis of array-CGH data in which the deletion break points cannot be accurately determined. Targeted testing for this variant in a larger population cohort will provide a more accurate estimation. Furthermore, screening of patients treated with FP-therapy may provide insights into a potential association between the identified deletion and 5-FU-induced toxicity.

## Conclusions

Our study reports a novel genomic deletion eliminating *DPYD* exons 9–10 which is associated with biochemical features of DPD deficiency. Given that DPD deficiency is a well-established cause of 5-FU-related toxicity, carriers of this CNV both at a heterozygote and homozygote state, are potentially at risk for experiencing severe adverse effects of FP treatment. The above raises the question whether preemptive genetic testing for DPD deficiency needs to be expanded through the inclusion of additional clinically relevant variants such as CNVs.

### Supplementary Information

**Supplementary Materials 1.**

## Data Availability

The variant reported in this study has been classified as likely pathogenic according to the joint consensus recommendation of the American College of Medical Genetics and Genomics (ACMG) and the Clinical Genome Resource (ClinGen) [44] and submitted to ClinVar with the accession number SCV004041586.

## Abbreviations

5-FU: 5-Fluorouracil
ACGS: Association for Clinical Genomic Science
ACMG: American College of Medical Genetics and Genomics
CES: Clinical Exome Sequencing
CNVs: Copy Number Variations
DPD: Dihydropyrimidine dehydrogenase
DSBs: Double-Strand DNA Breaks
EMA: European Medicines Agency
FAD: Flavin Adenine Dinucleotide
FMN: Flavin MonoNucleotide
FoSTeS: Fork Stalling and Template Switching
FPs: Fluoropyrimidines
INDELs: INsertions-DELetions
LINEs: Long Interspersed Nuclear Elements
MLPA: Multiplex Ligation-dependent Probe Amplification
MMRDR: Microhomology-Mediated Replication-Dependent Recombination
NADP^+^: Nicotinamide Adenine Dinucleotide Phosphate
NAHR: Non-Allelic Homologous Recombination
NGS: Next Generation Sequencing
NHEJ: Non-Homologous End Joining
PCR: Polymerase Chain Reaction
PharmVar: Pharmacogene Variation Consortium
SINEs: Short Interspersed Nuclear Elements
SNP: Single Nucleotide Polymorphism
SNVs: Single Nucleotide Variant
