# Supplementary material for: A novel large intragenic DPYD deletion causing dihydropyrimidine dehydrogenase deficiency: a case report
Source: BMC Med Genomics. 2024 Mar 25;17:78. doi: 10.1186/s12920-024-01846-2 (PMC10962175; doi:10.1186/s12920-024-01846-2)
Supplement: Supplementary file 1 — Supplementary Materials 1. [file 12920_2024_1846_MOESM1_ESM.docx]

**Table 1:** **Primers used in this study**

| **NAME** | **SEQUENCE** |
| --- | --- |
| DPYD_EX8_FW | ACA CTG GCT TTT CTT CTG CAT TT |
| DPYD_EX8_RV | ACA TCT TGC CGA AAT CTC TCC AT |
| DPYD_INT8_1F | TCA TTT GAA CCT GGA AGG GGT |
| DPYD_INT8_1R | TGA ATT ACA GTG GGC GGA GG |
| DPYD_INT8_2F | ACC AGA GGA CAC AAG ACA GAA |
| DPYD_INT8_2R | GCT TGT GTT GTG AGC CAG TT |
| DPYD_INT8_3F | GGC GTT CAT CCC CCA TCT AA |
| DPYD_INT8_3R | TAA CAG GCT TGA GGG CTT TCC |
| DPYD_INT8_4F | GGG GAT TAG GCG GTA GCA TC |
| DPYD_INT8_4R | TCC TTC CGC ATT AGC CTG AC |
| DPYD_INT8_5F | ACA AGC CCA ACT GCT ATG GT |
| DPYD_INT8_5R | CCA CAG ATC CCC TTA TGC CC |
| DPYD_INT8_6F | ATG CGT TAT GGA AGC AGG AC |
| DPYD_INT8_6R | CCA GTG TCC CCC TTT TCC TT |
| DPYD_INT8_7F | AGC TTG CAG TAT CTT GCC GA |
| DPYD_INT8_7R | CAC CCA ACT TCA CAA AAG GGC |
| DPYD_INT8_8F | GTA AGG CTG TAG CCA AGG TGA |
| DPYD_INT8_8R | CCC AAA GTG ATG GGG AGA TGA |
| DPYD_INT8_9F | AGG AGA CAA CAG TGA AGG GTC |
| DPYD_INT8_9R | CGC TTT CTG ACC CTT GGT TTC |
| DPYD_INT10_1F | AGC CCC CAT AAG CTG ACA AA |
| DPYD_INT10_1R | GCC TTC CTG TCC AGA TTC CA |
| DPYD_INT10_2F | ACC AGT CTT GAT GCC CTG TC |
| DPYD_INT10_2R | CTT TGT CTC CGT GCT GTG GT |
| DPYD_INT10_3F | GTG TCA TCC CCC TGT CAT CA |
| DPYD_INT10_3R | TCA ACA TGC CTC ACT GGT CTG |
| DPYD_INT10_4F | CTC CTT TAC CCT TCC ACA CCC |
| DPYD_INT10_4R | TGG GCA TTT CAC CTA GCA GT |
| DPYD_EX11_FW | ATG CTT GTT TCG CTG TCC TCA |
| DPYD_EX11_RV | GCT CCC AGC ACT GTA CCT TTA G |
| DPYD_INT8_1FSEQ | TTT CTG TTA ACT GGC TCA CAA CAC |
| DPYD_INT8_2FSEQ | TGG ATC TGG GAA GCT GGA TG |
| DPYD_INT8_3FSEQ | TTT CAT CTG CTG CTG ATT GTG |
| DPYD_INT8_4FSEQ | TGG CTC CTA ATG AGT CAG TAA G |
| DPYD_INT10_1RSEQ | CTA GGA AGT TCT TGT ATG GAA TCC |
| DPYD_INT10_2RSEQ | GGA GGG TAA AGG AAG AGG CAT C |
| DPYD_INT10_3RSEQ | GGG TTT CAC TGT GTT AGC CAG |
| DPYD_INT10_4RSEQ | GTC TAT CAT TGT TGG ACA TTT GGG |
| DPYD_INT10_5RSEQ | TTT AGA CAT GAA GTC CTT GCC C |

**Table 2:** **RepeatMasker output results**

| **SW score** | **Perc div.** | **Perc del.** | **Perc ins.** | **Query sequence*** | **Position in query** | | | **Matching**  **repeat** | **Repeat class/family** | **Position in repeat** | | | **ID** |
| --- | --- | --- | --- | --- | --- | --- | --- | --- | --- | --- | --- | --- | --- |
|  |  |  |  |  | Begin | end | (left) |  |  | Begin | end | (left) |  |
| 791 | 26.9 | 5.9 | 1.8 | Upstream_INTR8 | 1 | 276 | (724) | HAL1 | LINE/L1 | 103 | 390 | (2117) | 1 |
| 15 | 0.0 | 10.7 | 0.0 | Downstream_INTR8 | 79 | 106 | (894) | (AAGCAA)n | Simple _repeat | 1 | 31 | (0) | 2 |
| 1932 | 15.5 | 4.1 | 6.0 | Downstream_INTR8 | 522 | 891 | (109) | THE1B | LTR/ERVL-MaLR | 1 | 363 | (1) | 3 |
| 14 | 27.9 | 1.4 | 4.4 | Downstream_INTR8 | 899 | 968 | (32) | (ATTTTT)n | Simple _repeat | 1 | 68 | (0) | 4 |
| 228 | 28.6 | 5.6 | 1.2 | Upstream_INTR10 | 26 | 110 | (890) | L2c | LINE/L2 | 3105 | 3193 | (194) | 5 |
| 12 | 19.1 | 2.6 | 2.6 | Upstream_INTR10 | 155 | 192 | (808) | (TACTTAA)n | Simple _repeat | 1 | 38 | (0) | 6 |
| 3157 | 3.2 | 0.5 | 0.3 | Upstream_INTR10 | 423 | 437 | (563) | L1PA4 | LINE/L1 | (1) | 6154 | 6138 | 7 |
| 26 | 0.0 | 0.0 | 0.0 | Upstream_INTR10 | 438 | 462 | (538) | (TTA)n | Simple _repeat | 1 | 25 | (0) | 8 |
| 3157 | 3.2 | 0.5 | 0.3 | Upstream_INTR10 | 463 | 823 | (177) | L1PA4 | LINE/L1 | (18) | 6137 | 5777 | 7 |
| 1434 | 4.9 | 0.0 | 0.5 | Upstream_INTR10 | 824 | 1000 | (0) | L1P1 | LINE/L1 | 4981 | 5157 | (989) | 9 |
| 4381 | 2.4 | 1.1 | 0.2 | Downstream_INTR10 | 1 | 611 | (389) | L1P1 | LINE/L1 | 5158 | 5772 | (383) | 10 |
| 21 | 31.0 | 0.0 | 0.0 | Downstream_INTR10 | 740 | 802 | (198) | GA-rich | Low_complexity | 1 | 63 | (0) | 11 |

*INT8: breakpoint in intron 8, INT10: breakpoint in intron 10

**SW:** Smith-Waterman score of the match, **Perc div.:** % substitutions in matching region compared to the consensus, **Perc del.:** % of bases opposite a gap in the query sequence (deleted bp), **Perc ins.:** % of bases opposite a gap in the repeat consensus (inserted bp), **Query sequence:** name of query sequence, **Position in query:** starting position of match in query sequence, ending position of match in query sequence, no. of bases in query sequence past the ending position of match, **Matching repeat:** name of the matching interspersed repeat, **Repeat class/family:** the class of the repeat, **Position in repeat:** starting position of match in database sequence (using top-strand numbering), ending position of match in database sequence, no. of bases in (complement of) the repeat consensus sequence prior to beginning of the match.
